# *Francisella tularensis *subsp. *novicida *isolated from a human in Arizona

**DOI:** 10.1186/1756-0500-2-223

**Published:** 2009-11-06

**Authors:** Dawn N Birdsell, Tasha Stewart, Amy J Vogler, Elisabeth Lawaczeck, Alisa Diggs, Tammy L Sylvester, Jordan L Buchhagen, Raymond K Auerbach, Paul Keim, David M Wagner

**Affiliations:** 1 Center for Microbial Genetics and Genomics, Northern Arizona University, Flagstaff, AZ 86011-4073, USA; 2 Maricopa County Department of Public Health, Phoenix, AZ 85012, USA; 3 Arizona Department of Health Services, Phoenix, AZ 85007, USA; 4 Translational Genomics Research Institute, Phoenix, AZ 85004, USA; 5 Program in Computational Biology and Bioinformatics, Yale University, New Haven, CT 06520, USA

## Abstract

**Background:**

*Francisella tularensis *is the etiologic agent of tularemia and is classified as a select agent by the Centers for Disease Control and Prevention. Currently four known subspecies of *F. tularensis *that differ in virulence and geographical distribution are recognized:*tularensis *(type A), *holarctica *(type B), *mediasiatica*, and *novicida*. Because of the Select Agent status and differences in virulence and geographical location, the molecular analysis of any clinical case of tularemia is of particular interest. We analyzed an unusual *Francisella *clinical isolate from a human infection in Arizona using multiple DNA-based approaches.

**Findings:**

We report that the isolate is *F. tularensis *subsp. *novicida*, a subspecies that is rarely isolated.

**Conclusion:**

The rarity of this *novicida *subspecies in clinical settings makes each case study important for our understanding of its role in disease and its genetic relationship with other *F. tularensis *subspecies.

## Background

Tularemia is an incapacitating disease that presents in two main forms: pneumonic or ulceroglandular. The causative agent is the bacterium *Francisella tularensis*. Without antibiotic treatment, death occurs in 30%-60% of victims with the pneumonic form [1]. Because of this high mortality and its low infectious dose, *F. tularensis *was historically targeted for biological weapons development by the governments of the United States, Japan, and the former Soviet Union [1]. As a result, it is currently classified as a Category A Select Agent by the Centers for Disease Control and Prevention (CDC). Small animals (*e.g*., lagomorphs and rodents) may serve as hosts for *F. tularensis *and arthropods can serve as biological vectors for transmission. Although humans commonly acquire the infection through insect bites or handling of infected tissue, direct contact with environments where *F. tularensis *persists can comprise another source of infection [2].

Although a new species, *F. piscicida*, was recently identified [3], the three most commonly studied species within the *Francisella *genus are *F. tularensis*, *F. novicida*, and *F. philomiragia *[2]. *F. tularensis *is officially divided into three subspecies: *tularensis *(type A), which is further divided into subpopulations A.I and A.II [4,5]; *holarctica *(type B), and *mediasiatica *[2]. According to Staples *et al*. [6], these subspecies and subpopulations differ in pathogenicity, with *F. tularensis *subsp. *tularensis *subpopulation A.I more virulent than *F. tularensis *subsp. *holarctica*, which is more virulent than *F. tularensis *subsp. *tularensis *subpopulation A.II. *F. tularensis *subsp. *mediasiatica *has been reported to have virulence similar to *F. tularensis *subsp. *holarctica *[2], but was not included in the aforementioned study [6]. The rarely isolated *F. novicida *appears less virulent than *F. tularensis *[2], but has been shown to cause disease [7-10]. *F. novicida *is also genetically similar to *F. tularensis*, causing many to classify it as a fourth subspecies of *F. tularensis *[2,5,11,12], which this study also will do. *F. philomiragia *also causes human disease, usually in victims of near-drowning or immunocompromised individuals [8,13].

In addition to these recognized species and subspecies, PCR-based analyses of environmental samples have detected *Francisella*-like organisms in air, water, and soil [14,15]. These findings indicate the existence of a diverse group of organisms that are not well accounted for in the current taxonomy [2]. These *Francisella*-like organisms may or may not cause disease [14], though a recent report of two *Francisella*-like organisms from human infections with 16S rDNA sequences similar to those of the environmental *Francisella*-like organisms suggests the potential for human infection [16]. The deeper phylogenetic structure of the *Francisella *genus remains poorly understood but will improve as more detailed phylogenetic information is obtained from additional genomic sequences [2].

Because of pathogenicity differences, as well as the Select Agent status of *F. tularensis *(including *F. tularensis *subsp. *novicida*), correctly identifying clinical isolates as *F. tularensis *and assigning them to particular subspecies and/or molecular groups within *F. tularensis *is desirable. Biochemical-based subtyping methods are not ideal because of their labor intensiveness and because the results often are non-definitive. DNA-based assays provide the most robust means of accomplishing this task [2]. Molecular assays capable of assigning unknown isolates to *F. tularensis *subsp. *tularensis *or *holarctica *have been developed [17-19], but less attention has been paid to *F. tularensis *subsp. *novicida*.

The aim of this study was to determine the species and subspecies of a *Francisella *clinical isolate obtained from a human infection in Arizona. This aim was accomplished using multiple DNA-based approaches. We report that the clinical isolate has the molecular signature of *F. tularensis *subsp. *novicida*. Human disease events caused by *F. tularensis *subsp. *novicida *are rare, making this isolate of particular interest.

## Results and Discussion

### Clinical presentation

In August of 2006, a 15-year-old male who had presented with swelling on alternating sides of the face and neck over the preceding two to three weeks reported to a hospital in Maricopa County, Arizona. The patient, who had prior treatment with penicillin with no relief of symptoms, had no fever and normal white blood counts. The patient was negative on screening tests for EBV, bartonellosis, toxoplasmosis, tuberculosis, and coccidioidomycosis; blood culture also was negative. The patient was admitted for continued monitoring and an infectious disease consult, and he was treated with ampicillin and clindamycin. Biopsies in the area of the right parotid gland revealed a reactive lymph node. A biopsy sample was obtained for examination by microscopy, culture, and molecular typing. Based on the results of culture (next section), antibiotic treatment for the patient was switched to doxycycline even as the patient was on his way to recovery. No risk factors for tularemia acquisition were identified during interviews of the patient and his mother.

Hospital records were examined for this patient and it was determined that he had presented three times to a local emergency room in February 2006. On first visit, the patient reported intermittent malaise for 3-4 days, myalgia, diffuse abdominal pain, nausea, vomiting, and one episode of diarrhea. Elevated WBC was noted and pleural effusion and prominent mesenteric lymph nodes were observed on CT scan. The patient was diagnosed with pneumonia and was sent home with a prescription for zithromax. Four days later, the patient returned to the emergency room with complaints of shortness of breath and left shoulder and chest pain, although his previous symptoms had resolved. Pleural effusion was noted on chest radiographs. The patient was treated with rocephin, sent home with a prescription for augmentin, and told to continue with zithromax. Two weeks later the patient visited a local ER again with chief complaint of hand and foot pain and was advised to take ibuprofen.

The patient and his mother were interviewed with regards to previous medical history, travel, and other risk factors for tularemia, but none were revealed. The patient reported no travel history outside of county of residence (Maricopa) for several years, no contact with any animals or unchlorinated water, no hunting, and no insect bites. The family, which lives in an urban setting, owns three dogs but reported no tick problems. A sibling previously owned a ferret which was given away and later died. However, the patient had no contact with the ferret, and no handlers of the ferret reported illness. The family reported that rats had been observed outside on the family's property. The patient reported that he mows the lawn for a neighbor, but denied running over any animal carcasses and reported that he otherwise prefers to stay indoors.

### Initial identification

On culture, a light growth of Gram-negative rods was reported by a commercial lab, and was determined by biochemical tests to resemble *Kingella *species. Initial PCR analysis assigned the clinical isolate to *F. tularensis*. This finding was further confirmed by direct fluorescent antibody staining. The CDC in Fort Collins, CO, USA subsequently tested the isolate and reported that it was a "*F. tularensis *non-A, non-B subspecies."

### Molecular analyses

**Figure 1:**
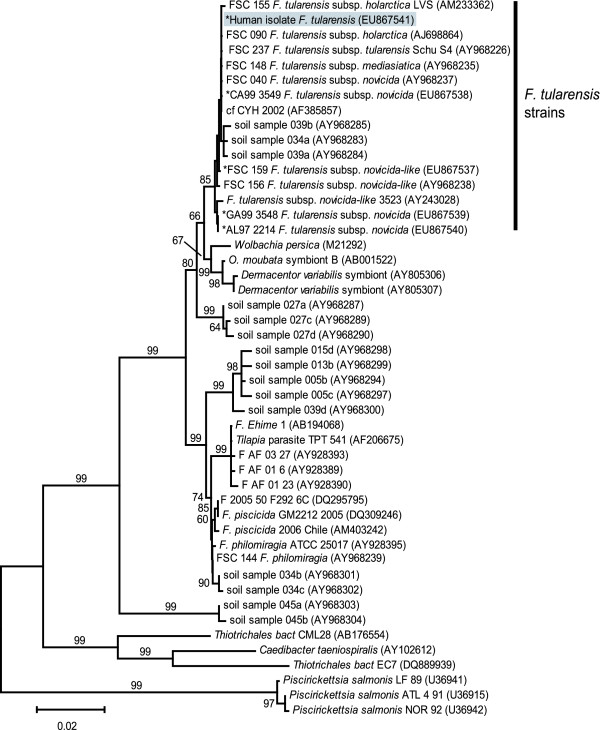
**Neighbor-joining phylogeny constructed from 16S rDNA gene sequences**. Sequences were obtained from the clinical human isolate, other *F. tularensis *isolates, and isolates from related species. The tree, which was created using MEGA2 software [23], is midpoint rooted. Bootstrap values ≥ 60 are presented and are based upon 1,000 replicates. The human clinical isolate clusters with the *F. tularensis *strains; right vertical bar indicates *F. tularensis *clade. Sequences with an asterisk (*) were generated as part of this study. All other sequences were obtained from GenBank. Accession numbers for all sequences are shown in parentheses. The scale bar, expressing mean character difference, corresponds to 0.02 substitutions per nucleotide position.

**Figure 2:**
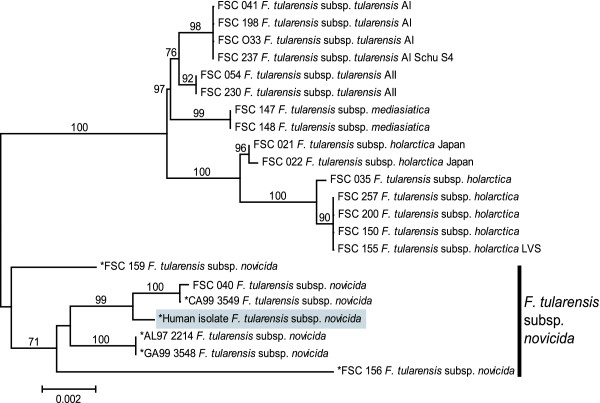
**Neighbor-joining phylogeny constructed from concatenated MLST gene fragment sequences**. Sequences were obtained from the clinical human isolate and other strains representing the four subspecies of *F. tularensis*. The tree, which was created using MEGA2 software [23], is midpoint rooted. Bootstrap values ≥ 60 are presented and are based upon 1,000 replicates. The human clinical isolate clusters with the *F. tularensis *subsp. *novicida *clade, which is indicated with the right vertical bar. Sequences with an asterisk (*) were generated as part of this study. All other sequences were obtained from GenBank and originated from a study by Svensson *et al*. [12]. The scale bar, expressing mean character difference, corresponds to 0.002 substitutions per nucleotide position.

Due to a lack of risk factors and the Select Agent status of *F. tularensis*, identifying the subspecies of this isolate was desirable. Because the isolate was neither of the two major *F. tularensis *subspecies, *tularensis *or *holarctica*, subspecies identification became of additional scientific interest. One possibility was that the isolate was not actually *F. tularensis*, but rather a closely-related but undefined *Francisella *species. To test this possibility, we subtyped the clinical isolate using multiple DNA-based approaches that provided increasing levels of resolution. Previous studies have indicated that analysis of the 16S rDNA gene sequence is useful for examining the relationship of *Francisella *to other closely-related genera [2,20]; this gene fragment also can be amplified from unculturable near-neighbor species [2]. Our analysis of the 16S rDNA gene confirmed that the clinical isolate was a member of the *F. tularensis *group (Fig 1). Multi-locus Sequence Typing (MLST), which is effective at assigning unknown isolates of *F. tularensis *to one of the four subspecies [12], indicated that the clinical isolate clustered within the subspecies *novicida *clade; this clade was supported by high bootstrap values in the phylogenetic analysis (Fig 2). Within the *novicida *clade, the clinical isolate possessed a unique MLST genotype (Fig 2). Barns *et al*. [14] previously reported that primer sets targeting a putative *succinate dehydrogenase *locus were useful for discriminating among the subspecies of *F. tularensis*. Sequencing analysis of the amplicon generated with their primer sets indicated the clinical isolate was identical to that of several *F. tularensis *subspecies *novicida *isolates (data not shown). Finally, variable-number tandem repeat analysis utilizing 11 loci [21] grouped the clinical isolate with other *F. tularensis *subspecies *novicida *isolates (data not shown).

## Conclusion

We conclude that the clinical isolate from this human case belongs to *F. tularensis *subsp. *novicida*. It is unclear if the hospitalization for abdominal pain and pneumonia six months earlier was related to the subsequent diagnosis of tularemia made from the lymph node biopsy. However, because this subspecies is known to exhibit low virulence, these findings suggest that this case may have been a lingering, chronic infection. Despite the fact that this subspecies is infrequently associated with disease in immuno-competent hosts, several cases have been documented [7-10] and *F. tularensis *subsp. *novicida *is considered a human pathogen and a Select Agent. Because infections by *F. tularensis *subsp. *novicida *may often lack clinical significance, the frequency of human infections may be underreported [7,10,22]. The rarity of this *novicida *subspecies in clinical settings makes each case study important by advancing our understanding of its role in disease and its genetic relationship with other *F. tularensis *subspecies.

## Methods

### 16S rDNA and MLST Gene Sequencing

Initial amplification of the 16S rDNA gene was performed using the forward primer 27F.1G (5'-GAGRGTTTGATCMTGGCTCAG-3') and two reverse primers, 787Rb (5'-GGACTACNRGGGTATCTAAT-3') and 1391-R (5'-GACGGGCGGTGTGTRCA-3'). Amplification was carried out in 10 μl reactions containing 1× PCR buffer (Invitrogen, Carlsbad, CA), 2 mM MgCl_2 _(Invitrogen), 0.2 mM each deoxynucleoside triphosphate (Invitrogen), 0.8 U Platinum *Taq *polymerase (Invitrogen), and 1 μl DNA template. Thermal cycling conditions were 94°C for 5 min, 35 cycles of 94°C for 30 s, 55°C for 30 s and 72°C for 1.5 min, and a final extension of 72°C for 5 min. Multi-locus sequence typing (MLST) genes were amplified as described previously [12]. Sequencing of these 16S rDNA and MLST gene amplicons was carried out on an ABI 3100 (Applied Biosystems, Foster City, CA) using BigDye terminator cycle sequencing reagents (v. 3.1 Applied Biosystems).

### Nucleotide sequence accession numbers

Nucleic acid sequences generated for this study were deposited in GenBank. Accession numbers for 16S rDNA sequences are as follows: Human isolate *F. tularensis *[GenBank:U867541], CA99 3549 *F. tularensis *subsp. *novicida *[GenBank:U867538], FSC 159 *F. tularensis *subsp. *novicida-*like [GenBank:U867537], GA99 3548 *F. tularensis *subsp. *novicida *[GenBank:U867539], AL97 2214 *F. tularensis *subsp. *novicida *[GenBank:U867540]. Accession numbers for MLST gene fragment sequences are as follows: *uup*: [GenBank:U867519 to U867524]; *aroA*: [GenBank:U867525 to U867530]; *atpA*: [GenBank:U867531 to U867536]; *pgm*: [GenBank:U867548 to U867553]; *tpiA*: [GenBank:U867554 to U867559]; *trpE*: [GenBank:U867560 to U867565]; *parC*: [GenBank:U867542 to U867547] for isolates FSC 156, FSC 159, CA99 3549, GA99 3548, AL97 2214, and the human isolate, respectively.

## Competing interests

The authors declare that they have no competing interests.

## Authors' contributions

DNB carried out the molecular genetic studies, participated in the construction of the phylogenetic trees and drafted the manuscript. JLB carried out the molecular genetic studies. AJV participated in the design of the study and drafted the manuscript. TS, EL, AD, and TLS participated in the clinical study. RKA participated in the computational *in silico *data analysis. PK participated in data interpretation and drafted the manuscript. DMW assisted in the design of the study and drafted the manuscript. All authors read and approved of the final manuscript.

## Authors' information

DNB, Ph.D., Northern Arizona University, Flagstaff, Arizona

TS, MPH, Maricopa County Department of Public Health, Phoenix, Arizona

AJV, Ph.D., Northern Arizona University, Flagstaff, Arizona

EL, DVM, Arizona Department of Health Services, Phoenix, Arizona

AD, MPH, Maricopa County Department of Public Health, Phoenix, Arizona

TLS, BSN, Maricopa County Department of Public Health, Phoenix, Arizona

JLB, Northern Arizona University, Flagstaff, Arizona

RKA, M.S., Northern Arizona University, Flagstaff, Arizona

PK, Ph.D., Northern Arizona University, and Translational Genomics Research Institute, Flagstaff, Arizona

DMW*, Ph.D., Northern Arizona University, Flagstaff, Arizona
